# Parents’ preferences for vaccinating daughters against human papillomavirus in the Netherlands: a discrete choice experiment

**DOI:** 10.1186/1471-2458-14-454

**Published:** 2014-05-15

**Authors:** Robine Hofman, Esther W de Bekker-Grob, Hein Raat, Theo JM Helmerhorst, Marjolein van Ballegooijen, Ida J Korfage

**Affiliations:** 1 Department of Public Health Erasmus MC, University Medical Centre, Rotterdam, P.O. Box 2040 3000, CA, The Netherlands; 2 Department of Obstetrics and Gynaecology Erasmus MC, University Medical Centre, Rotterdam, P.O. Box 2040 3000, CA, The Netherlands

**Keywords:** Preferences, Human Papillomavirus, Vaccination, Discrete choice experiment, Parents

## Abstract

**Background:**

To generate knowledge about potential improvements to human papillomavirus (HPV) vaccination information and organization strategies, we assessed how aspects of HPV vaccination are associated with parents’ preferences for their daughters’ uptake, and which trade-offs parents are willing to make between these aspects.

**Methods:**

A discrete choice experiment (DCE) was conducted among parents with a daughter aged 10–12 years. Panel mixed logit regression models were used to determine parents’ preferences for vaccination. Trade-offs were quantified between four vaccination programme aspects: degree of protection against cervical cancer, duration of protection, risk of serious side-effects, and age of vaccination.

**Results:**

Total response rate was 302/983 (31%). All aspects influenced respondents’ preferences for HPV vaccination (p < 0.05). Respondents preferred vaccination at age 14 years instead of at a younger age. Respondents were willing to trade-off 11% of the degree of protection to obtain life-time protection instead of 25 years. To obtain a vaccination with a risk of serious side-effects of 1/750,000 instead of 1/150,000, respondents were willing to trade-off 21%.

**Conclusions:**

Uptake may rise if the age ranges for free HPV vaccinations are broadened. Based on the trade-offs parents were willing to make, we conclude that uptake would increase if new evidence indicated outcomes are better than are currently understood, particularly for degree and duration of protection.

## Background

Human Papillomavirus (HPV) infection is a necessary factor in the development of cervical cancer [1]. Vaccines are available against HPV 16 and 18, that are responsible for 71% of all cervical cancers worldwide [2]. Most European countries offer the vaccine to girls aged between 9 to 17 years [3,4]. However, uptake rates vary considerably between countries (range 17-84%) [4]. In 2009, the Dutch National Immunization Programme (NIP) was extended to include the bivalent HPV vaccine for 12-year-old girls. A catch-up campaign was organized in 2009 for 13 to 16 year old girls. The uptake rate of this campaign was 52% [5]. In 2010, 56% of all 12-year-old girls were vaccinated against HPV and in 2011 the uptake was 58% [6]. Vaccination through the NIP is voluntary, is free of costs, and 12-year-olds are legally entitled to make their own decision about uptake.

The attitude of parents and girls towards HPV vaccination and consequently its uptake, may be influenced by their perception of the advantages and disadvantages of the vaccine [7]. An advantage is the (partial) protection against cervical cancer that the vaccine provides. The fact that only partial protection is provided by HPV vaccinations may be considered a disadvantage: in spite of HPV vaccinations, girls are still vulnerable to develop cervical cancer [8]. Furthermore, insecurity about the safety of the vaccine [8-11], anticipated side-effects such as pain or discomfort [10,12], and cost [11,13,14] can be considered as disadvantages. Parents and girls may become ambivalent towards HPV vaccination when they weigh these ‘pros’ and ‘cons’ [8], and have, e.g., simultaneous positive and negative evaluations of an attitude object [15,16], in this case HPV vaccination. This may lead to postponing decisions about uptake, and hence, low uptake rates, while a proportion of girls (and parents) potentially had decided to have the HPV vaccination if better information had been available to them. Our study aimed to generate knowledge to improve information and organization strategies. We therefore wanted to assess which vaccine characteristics were important for parents and girls when deciding about uptake and which trade-offs they were willing to make between these characteristics. We used a discrete choice experiment (DCE), a quantitative approach that is increasingly used in health care to elicit preferences [17,18]. Although Dutch girls are legally allowed to decide about their own uptake, previous research showed that twelve to thirteen year old girls made a shared decision with their parents regarding uptake [19]. We therefore aimed at assessing preferences for HPV vaccination in both girls [20] and in parents. The current study describes the DCE as conducted among parents.

## Methods

### Discrete choice experiment

In DCEs, it is assumed that a medical intervention, such as a vaccination programme, can be described by its characteristics (attributes; e.g. duration of protection) [21], and by variants of that characteristic (levels of the attribute; e.g. a duration of protection of 6 years, 25 years and life-time). Furthermore, it is assumed that individual preferences for a medical intervention are determined by the levels of those attributes [21]. The relative importance of attributes and the trade-offs that respondents make between them can be assessed by offering a series of choices between two or more medical intervention alternatives with different combinations of attribute levels (Table 1) [22]. A DCE is a straightforward task, which more closer resembles a real world decision (i.e. trading off health and non-health outcomes) in comparison with other stated preference techniques [23]. We conducted the DCE according to the International Society for Pharmacoeconomics and Outcomes Research (ISPOR) DCE guideline [24] and Lancsar and Louviere’s guide [25].

**Table 1:** Choice set example

**Attributes**	**Programme A**	**Programme B**	**No vaccination**
Degree of protection against cervical cancer	70%	90%	0%
Duration of protection	Lifetime	6 years	n.a.
Risk of serious side-effects	1:750,000	1:750,000	No risk
Age at vaccination	14 years	9 years	n.a.
**Which vaccination programme do you prefer?**	• **A**	• **B**	• **None**

n.a. = not applicable.

### Attributes and attribute levels

The selection of attributes and their levels was based on data from the literature [7]; focus groups with 36 parents about decisional strategies and factors that could guide HPV vaccination intentions [8]; and interviews with experts in the field of HPV vaccination, such as professors in gynaecology, adolescent public health, and infectious disease control (n = 8). This resulted in eight attributes which were ranked by parents (n = 10) and the experts (n = 8). The attributes identified as most relevant were: 1) the degree of protection against cervical cancer; 2) the duration of protection; 3) the risk of serious side-effects (e.g. hospitalization); and 4) the age of vaccination (Table 2). These were included in the DCE design. Attributes that were considered less relevant were total costs, the risk of mild side-effects, the reduction in required number of pap tests, and the HPV vaccine being recommended by e.g. one’s general practioner/the government/family or friends. Levels of the attributes were selected in such a way that they were plausible and relevant from both the clinical and the policy viewpoint. Levels of risk of serious side-effects were based on a report of the Centers for Disease Control and Prevention (CDC) (2009).

**Table 2:** Considered attributes and levels for HPV vaccination

**Attributes**	**Levels**
Degree of protection against cervical cancer (%)	50, 70, 90
Duration of protection (yrs)	6, 25, lifetime
Risk of serious side-effects (1 out of…)	750,000, 150,000, 30,000
Age at vaccination (yrs)	9, 12, 14

### Study design

The combination of four attributes with three levels each resulted in 81 (3^4^) hypothetical HPV vaccination alternatives. Using an efficient design by maximizing D-efficiency (SAS software version 9.1, SAS Institute Inc., Cary, NC, USA), 54 choice sets were constructed to be able to estimate all main effects and all two-way interactions between attributes. Choice sets consisted of two HPV vaccination alternatives and a ‘no HPV vaccination’ option to allow respondents to ‘opt out’ (Table 1). HPV vaccination is a preventive medical intervention and, as in real life, respondents are not obliged to opt for HPV vaccination. Respondents were asked to consider all three options in a choice set as realistic alternatives and to choose the option that appealed most to them. Presenting a single individual with a large amount of choice sets is expected to result in a lower response rate and/or response reliability [26,27]. We therefore used a blocked design [22], which resulted in dividing the 54 choice sets over six questionnaires containing nine choice sets each.

### Study sample

A sample of parents with a daughter aged 10–12 years was approached through five primary school administrations in urban and rural areas in the Netherlands. These school administrations consisted of a total of 57 schools, of which 55 were willing to participate. Calculation of optimal sample sizes for estimating non-linear discrete choice models from DCE data is complicated as it depends on the true values of the unknown parameters estimated in the choice models [25]. One however rarely requires more than 20 respondents per parameter to estimate reliable models [25]. Our DCE contained 8 main-effect parameters (see equation 1). It, therefore, needed to include at least 160 respondents.. Taking into account some two-way interactions between attributes, 300 questionnaires was expected to be sufficient based on other studies [24,28,29].

### Questionnaire

The first page of the questionnaire provided information about HPV and its link with cervical cancer, and HPV vaccination. In the DCE section, respondents were asked to choose the option that appealed to them most. A separate sheet showed the percentages of the degree of protection illustrated with bar graphs, and a description of the risk of serious side-effects in words (i.e. the risk of serious side-effects is small, very small or extremely small).

We assessed respondents’ understanding of the DCE task by including a dominant choice set as a rationality test. In this choice set the HPV vaccine was given at the age of 12 years in both alternatives, while one alternative was characterized by logically preferable levels on all other attributes. Convergent validity was checked with a ranking task, i.e. ranking the four attributes of HPV vaccination from most important to least important. To gain more insight into respondents’ understanding of the DCE task, i.e. comparing risks and percentages, we included the Subjective Numeracy Scale (SNS), a scale that correlates well with objective measures of numeracy skills [30,31]. Higher scores indicate higher numeracy.

The questionnaire was pilot tested to check for face validity and for problems in interpretation (n = 16). This resulted in an improved explanation of the risk of serious side-effects. Approval for the study was obtained from the Medical Ethics Committee, Erasmus MC, University Medical Center Rotterdam (MEC 2008–206).

Questionnaires and information letters were sent to primary schools between March and June 2009 to be distributed to 10 to 12-year-old girls to give to their parents. Parents could return the questionnaire in a postage-paid envelope that was included in the mailing package.

### Statistical analyses

The DCE was analysed by taking each choice among the three options as an observation, i.e. two ‘no’ and one ‘yes’ responses. The observations were analysed by panel mixed logit regression models to take heterogeneity as well as correlation between the choice sets completed by each individual into account [22]. After testing for linear continuous effects of the attributes, we selected the model with the best fit based on the Akaike information criterion (AIC). Doing so, the following utility model was estimated:

(1) $$\begin{array}{ll} V=\beta_{0} & +\beta_{1}\mathrm{EFFECTIVENESS}+\beta_{2}\mathrm{DURATION}\_25Y\\ & +\beta_{3}\mathrm{DURATION}\_\mathrm{LIFETIME}\\ & +\beta_{4}\mathrm{SERIOUS}\_1/150,000\\ & +\beta_{5}\mathrm{SERIOUS}\_1/30,000+\beta_{6}\mathrm{AGE}\_12Y\\ & +\beta_{7}\mathrm{AGE}\_14Y \end{array}$$

V is the observable utility that is composed of the preference scores (β-coefficients) for the individual and the characteristics of the HPV vaccination alternative. β_1_-β_7_ are coefficients of the attributes indicating the relative weight individuals place on a certain attribute (level). When considering an HPV vaccination that generates a 50%, 70% or 90% protection rate, the coefficient β_1_EFFECTIVENESS should be multiplied five, seven or nine times, respectively. The statistical significance of a coefficient (p-value ≤0.05) indicates that respondents differentiated between one attribute (or attribute level) and another in making stated choices about HPV vaccination programmes. A priori, we expected all attributes to be significant. The sign of a coefficient reflects whether the attribute has a positive or negative effect on the preference score of HPV vaccination. We expected that only the attribute ‘risk of serious side-effects’ would have a negative effect. The value of each coefficient represents the relative importance respondents assign to an attribute (level).

Sensitivity analyses were conducted to explore the impact of excluding respondents who failed the rationality test by excluding their data from the sample and re-running the analysis [32,33]. Also a number of two-way interactions between attributes were added to the main effects model to test which ones were significant and improved the fit of the model.

**Figure 1:**
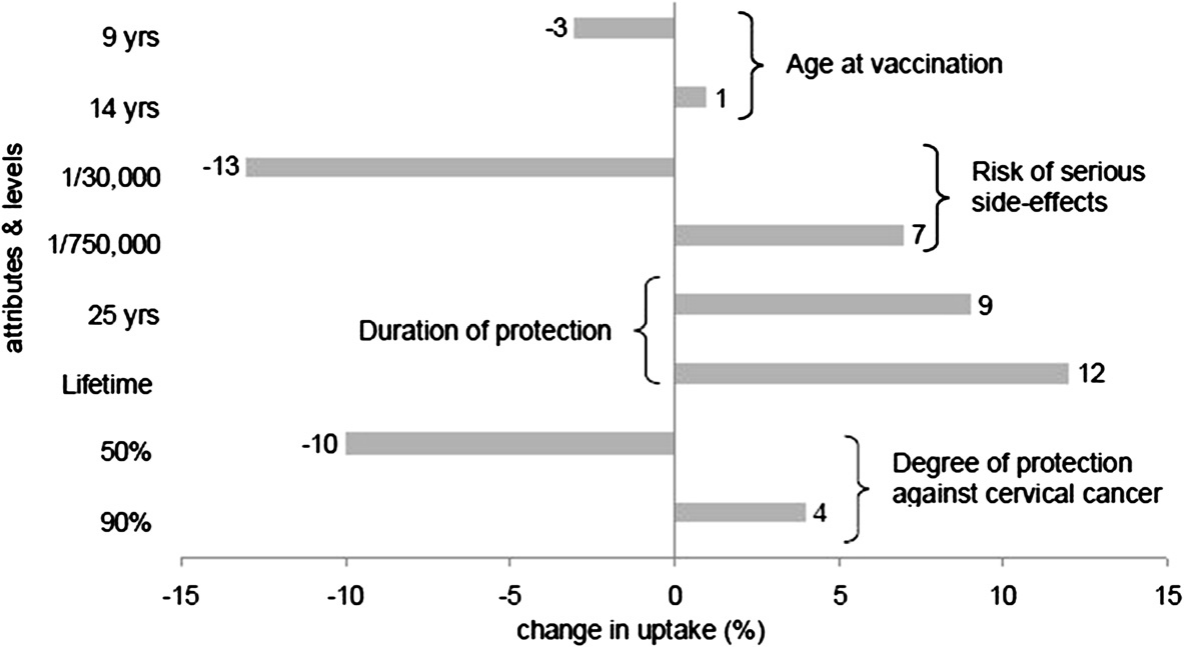
**Univariate marginal estimates for change in predicted probability of participation of parents; highest and lowest value for attributes versus base-case.** The base-case is an HPV vaccination at age 12 years, 1/150,000 risk of serious side-effects, duration of protection of 6 years, and 70% degree of protection against cervical cancer. This base-case is indicated as zero change in the probability of the x-axis. Assuming our base-case, the expected uptake was 63.3%

The trade-offs respondents were willing to make between the HPV vaccination attributes were calculated by the ratios of the coefficients of the different attributes with the degree of protection as the denominator. Choice probabilities for HPV vaccination uptake were also calculated to provide a way to convey DCE results to decision makers that is easier to interpret. The probability that an individual will say “yes” to an HPV vaccination programme is equal to:

(2) $$\;P=1/\left({1+e}^{‒V}\right)$$

where V is defined as in Equation 1. We calculated the choice probability (i.e. the mean uptake) of a base-case compared to no vaccination (V (no vaccination) = 0)). Our base-case represents an HPV vaccination programme at the age of 12 years, a 1/150,000 risk of serious side-effects, a duration of protection of 6 years, and a 70% degree of protection. This base-case was chosen to correspond with i) the Dutch situation (vaccination at the age of 12 years) and ii) an HPV vaccination programme that contained most plausible levels based on literature. Noteworthy, in the calculation of the mean uptake all heterogeneity of the respondents was taken into account as the mean uptake is not just equal to the uptake of someone with average coefficient values of the levels. We presented these results in a “tornado” graph to illustrate the marginal effect on uptake of varying one attribute level at a time from the base-case, holding all other attributes constant [34] (Figure 1).

## Results

### Respondents

The response rate was 302/983 (31%). In total, 294 out of these 302 parents (97%) completed the DCE task and were included for further analyses. The mean age of the parents was 42.7 (SD = 3.4) years. 90% of the respondents were female and about half had an intermediate educational level (54%) and had a religious affiliation (56%) (Table 3).

**Table 3:** Respondent characteristics

	**(n = 294)**	
**Characteristics**		
**Age (years) Mean (SD) range**	42.7 32-53	(3.4)
	**n**	**(%)**
**Sex**		
Female	264	(90.1)
**Educational level**		
Low	26	(9.3)
Intermediate	151	(53.9)
High	103	(36.3)
**Religion**		
None	128	(43.7)
Christian	156	(53.2)
Muslim	1	(0.3)
Other	8	(2.7)
**Country of birth**		
The Netherlands	272	(92.5)
**Country of birth of parents**		
Both parents in the Netherlands	268	(96.8)
One parent outside the Netherlands	3	(1.1)
Both parents outside the Netherlands	6	(2.2)
**Daughter HPV vaccinated**		
Yes	36	(12.4)
**Intention if daughter not vaccinated**		
Yes	146	(58.4)
No	54	(21.5)
Don’t know	51	(20.3)
**Job status**		
Paid job	259	(88.4)
Housewife or -man/unpaid job/student	28	(9.6)
No job	6	(2.0)
**Marital status**		
Married/cohabiting	274	(93.8)
Partner, but living alone	7	(2.4)
No partner	11	(3.8)
**Net income per month (euro’s)**		
< 1.500	13	(5.2)
1.500 – 3.000	113	(45.6)
3.000 – 4.500	86	(34.7)
> 4.500	36	(14.5)

### DCE results

All vaccine characteristics proved to influence parents’ preferences for HPV vaccination (p < 0.05; Table 4). The directions of the coefficients of the characteristics were in accordance with our priori hypotheses, indicating theoretical validity. The positive directions of the coefficients ‘degree of protection’ and ‘duration of protection’ indicated that parents preferred a higher protection rate and a longer duration of protection over a lower protection rate and a shorter duration of protection. The negative direction of serious side-effects indicated that parents preferred an HPV vaccination programme with low levels of serious side-effects. Most estimated standard deviations were significant, which indicated preference heterogeneity among respondents for several characteristics of HPV vaccination. Parents did not prefer vaccination at age 12 years over vaccination at the age of 9 years, but did prefer vaccination at age 14 years over vaccination at age 9 years (Table 4).

**Table 4:** Respondents’ preferences for HPV vaccination based on a panel mixed logit model [N = 294]

**Attributes**		**Coefficient**		
		**Value**		**(95% CI)**
Constant (vaccination)	Mean	-3.18	***^a^	(-4.50 to -1.86)
	S.D.	9.26	***	(7.62 to 10.9)
Degree of protection against cervical cancer (per 10%)	Mean	1.18^b^	***	(0.99 to 1.36)
	S.D.	0.75	***	(0.60 to 0.90)
Duration of protection 6 years (omitted)^c^	Mean	-2.37	***	(-2.72 to -2.03)
	S.D.	1.46	***	(1.41 to 1.51)
Duration of protection 25 years	Mean	0.56	***	(0.40 to 0.72)
	S.D.	0.36	***	(0.10 to 0.62)
Duration of protection lifetime	Mean	1.81	***	(1.51 to 2.11)
	S.D.	1.42	***	(1.14 to 1.70)
1/750,000 risk of serious side effects (omitted)^c^	Mean	3.04	***	(2.54 to 3.55)
	S.D.	3.18	***	(2.98 to 3.38)
1/150,000 risk of serious side effects	Mean	0.62	***	(0.37 to 0.86)
	S.D.	0.70	***	(0.42 to 0.98)
1/30,000 risk of serious side effects	Mean	-3.66	***	(-3.06 to -4.25)
	S.D.	3.11	***	(2.45 to 3.77)
Vaccination at age 9 years (omitted)^c^	Mean	-0.65	***	(-0.86 to -0.44)
	S.D.	0.32	***	(0.30 to 0.33)
Vaccination at age 12 years	Mean	0.11		(-0.06 to 0.29)
	S.D.	0.29	***	(0.10 to 0.48)
Vaccination at age 14 years	Mean	0.54	***	(0.36 to 0.72)
	S.D.	0.29	***	(0.10 to 0.48)
**Model fits**				
Log-Likelihood function			-1205.62	
Akaike information criterion			0.93	
Bayesian information criterion			0.96	
Pseudo R-squared			0.58	

*Notes:* Effects coded variables used for protection duration, serious side effects, and age at vaccination; Normal distribution for random coefficients used on all attributes; Number of observations = 7938 (i.e. 294 respondents completed 9 choice sets containing 3 response options each).

^a^***Denotes p < .01; ^b^When looking at an HPV vaccination that generates a 50%, 70% or 90% protection rate, the coefficient should be multiplied five, seven or nine times; ^c^The value of the omitted term equals the negative sum of the coefficients.

Sensitivity analyses showed that excluding the data of five out of 294 parents (1.7%) who ‘failed’ the rationality test had no relevant impact on the size or relative importance of the attributes. Adding two-way interactions did not significantly improve the fit of the model (data not shown).

### Ranking test and numeracy

The results of the ranking task showed that the most important attributes were: the degree of protection (49%; 95% CI: 0.42 to 0.54); the risk of serious side-effects (44%; 95% CI: 0.38 to 0.50); and the duration of protection (5%; 95% CI: 0.03 to 0.09). These results are in accordance with the DCE results (i.e. the order of importance is the same as the order of the coefficients), supporting a convergent validity of the DCE results.

Parents’ scores on the Subjective Numeracy Scale ranged from 1.5 to 6.0 with a median of 4.6 (95% CI: 4.50 to 4.75, calculated with bootstrapping) and inter quartile range (IQR) of 1.2 (data negatively skewed). The Cronbach alpha coefficient was 0.88, suggesting very good internal consistency reliability.

### Trade-offs

Parents were willing to trade-off the degree of protection against cervical cancer in order to gain improvement in the levels of the other attributes. They were willing to trade-off 11% of the degree of protection to obtain life-time protection instead of 25 years. To obtain an HPV vaccination with a risk of serious side-effects of 1/750,000 instead of 1/150,000, parents were willing to trade-off 21%. To get a vaccination at age 14 years instead of 9 years, parents were willing to trade-off 10% (Table 5).

**Table 5:** Respondents’ trade-offs between degree of protection and different aspects of a vaccination programme

**Change in levels**	**Trade-off in a decreased degree of protection against cervical cancer (%; CI)**
A **protection duration** of lifetime instead of 25 years	10.7 (8.6 to 12.7)
A **risk of serious side-effects** of 1/750,000 instead of 1/150,000	20.6 (15.9 to 25.3)
A vaccination at **age** 14 years instead of 9 years	10.1 (8.3 to 11.9)

### Expected uptake of HPV vaccination

Assuming our base-case HPV vaccination programme (an HPV vaccination programme at the age of 12 years, a 1/150,000 risk of serious side-effects; a duration of protection of 6 years, and a 70% degree of protection), the expected uptake based on parents’ preferences was 63.3%. Especially an increase in the duration of protection from 6 years to lifetime would result in a relatively large increase in the expected uptake (12.2%). On the other hand, an increased risk of serious side-effects from 1/150,000 to 1/30,000 would result in a decrease in the expected uptake (13.4%) (Figure 1).

## Discussion

This study shows that the degree of protection against cervical cancer, the duration of protection, the risk of serious side-effects, and the age of vaccination, significantly influenced parents’ preferences for HPV vaccination. Parents preferred vaccination at age 14 years over age 9 years. Although parents preferred a higher degree of protection against cervical cancer, they were willing to trade-off some degree of protection in order to gain improvement in the levels of the other attributes.

Our finding that the duration and the degree of protection were relevant for parents’ preferences for HPV vaccination was also found in a DCE study among mothers [35]. Our study is innovative, in that we used new attributes (risk of serious side-effects and age at vaccination), and that we sampled European parents. Also, our findings were in line with the findings of a DCE study, which investigated girls’ preferences for HPV vaccination [20]. With the exception that parents prefer vaccination at age 14 years instead of at a younger age, whereas girls prefer vaccination at age 12 years over age 14 years [20]. Our findings are consistent with the results from the previous studies which found that vaccine acceptability of parents increases as the proposed age of vaccination increases (infant, preadolescent and older teenagers) [10]. This might have implications for vaccination programmes: uptake may rise if the age ranges within which a girl is entitled to free HPV vaccinations are broadened to e.g. 12 to 16 years.

The expected uptake of our base-case HPV vaccination programme was 63% based on parents’ preferences. This rate is higher than the actual uptake of 52% in the Netherlands in 2009 at the time our study was conducted [5] and higher than the 58% of parents in our study who intend to have their daughter vaccinated, but lower than the uptake of other childhood vaccination in the Dutch NIP, which is 95% [36]. A possible explanation might be the current uncertainty considering several aspects of the vaccine. Our results showed that the unknown duration and degree of protection against cervical cancer and the unknown risk of serious side-effects all played an important role in parents’ choices about HPV vaccination uptake. If for example the duration of protection was lifetime instead of 6 years, the expected uptake would increase to 76-80%. To date, follow-up data on HPV vaccinated young women are available for 8.4 years [37]. Therefore the effects of HPV vaccination on the long-term are unknown. Furthermore, when the HPV vaccination campaign started in the Netherlands in 2009, an intensive societal debate involving politics, physicians, media, parents and girls was ongoing. Contradictions in this debate could also explain the low uptake. Possibly parents and girls became ambivalent towards HPV vaccination, i.e. they may have held simultaneous positive and negative feelings towards HPV vaccinating, which can have a moderating effect on attitude-intention and attitude-behavior relationships resulting in postponing the decision about uptake [38].

Our study has some limitations. First, the majority of responding parents were mothers, although the questionnaire was addressed to both parents. This seems common in studies assessing parental attitudes regarding HPV vaccination [10,39,40]. We do not expect this limitation to have biased the results. Second, the response rate of parents was relatively low (31%). However, the rate is similar to other DCE studies [28,41]. As indicated by the high educational level of most parents, due to the low response rate, our sample may not be representative of the general population. This may limit the external validity of our results. We recommend that in future research ways are sought to include parents with a low educational level in DCE studies. The relatively high score for subjective numeracy score indicates that our sample probably did understand the risks and percentages they had to compare in the DCE task.

## Conclusion

In conclusion, this study shows that parents’ preferences for HPV vaccination were influenced by the degree of protection against cervical cancer, the duration of protection, the risk of serious side-effects, and the age of vaccination. Uptake may rise if the age ranges within which a girl is entitled to free HPV vaccinations are broadened. Based on the trade-offs parents were willing to make, we conclude that uptake would increase if new evidence indicated outcomes are better than are currently understood, particularly for degree and duration of protection.

## Competing interests

The authors declare that they have no conflict of interests.

## Authors’ contributions

IJK conceived the idea for the study, designed the protocol, and supervised the execution of the study; EWBG, RH, IJK, MB, TJMH, and HR designed the questionnaire; RH performed the retrieval of the sample and was responsible for all mailings; RH and EWBG were responsible for the database design and data entry; EWBG performed the statistical design and analyses; RH drafted the report. All authors revised the article critically and approved the final version to be published.

## Pre-publication history

The pre-publication history for this paper can be accessed here:

http://www.biomedcentral.com/1471-2458/14/454/prepub
